# Community based distribution agents’ approach to provision of family planning information and services in five Nigerian States: A mirage or a reality?

**DOI:** 10.4102/phcfm.v3i1.228

**Published:** 2011-11-17

**Authors:** Mojisola Fayemi, Gloria Momoh, Oluwafemi Oduola, Grace Delano, Oladapo Ladipo, Olayimika Adebola

**Affiliations:** 1 Association for Reproductive and Family Health, Ibadan, Nigeria; 2 Emma Skipper Foundation, Ogun State, Nigeria

## Abstract

**Background:**

Reducing maternal mortality in Nigeria has received continuous attention both nationally and internationally.

**Objectives:**

This article highlights the outcome of an intervention which sought to address maternal mortality reduction through increasing contraceptive uptake in 10 rural local government areas (LGAs)in five Nigerian states.

**Method:**

The community based distribution (CBD) approach was used in the implementation of a three year intervention that targeted 10 LGAs. Two hundred and fifty community members were trained as community based distribution agents (CBDA) to provide information on reproductive health, provide non-prescriptive family planning (FP) commodities, treat minor aliment and make referrals to primary health centres within the communities.

**Results:**

Final evaluation revealed an increase in the proportion of community members who had utilised FP commodities at all, from 28% at baseline to 49%, and an increase in the proportion of current contraceptive users from 16% at baseline to 37%. An average of 50% increase in clientele patronage was also observed in the 10 LGAs’ primary health care centres.

Most (96%) of the interviewed CBDA agents reported that a drug-revolving system was in place to ensure that drugs and commodities were available. On-the-spot assessment of the service forms revealed that 86% of them had their activities regularly recorded in their worksheets. Some of the challenges faced by CBDA were discrimination and misconception of community members about family planning (38%), inadequate financial support (14%), and transportation problems (8%).

**Conclusion:**

This study has demonstrated that the CBD approach played a critical role in enhancing access to Reproductive Health and Family Planning information and services in the project communities.

## Introduction

### Setting

The actualisation of the United Nations Millennium Development Goals, especially with regard to maternal mortality reduction, is off target, particularly in sub-Saharan Africa.^1^ In Nigeria, the progress towards achieving this goal is slow, with a maternal mortality ratio of 545 per 100 000 live births.^2^ There is a connection with a plethora of factors such as socio-cultural aspects, poverty, malnutrition, lack of access to health facilities, weak health systems, deliveries by unskilled birth attendants and a low contraceptive uptake in the country. Several studies on fertility has demonstrated a strong relationship between high contraceptive use and reduced maternal mortality.^3, 4^ Despite considerable knowledge of contraceptive methods, Nigeria's contraceptive prevalence is low, ranging from 4% in the far north to 10% in the north central and 23% in the south-west areas, resulting in a total fertility rate (TFR) of 5.7.^2^ This fertility rate is higher amongst populations in the rural areas in Nigeria.

One of the services expected to be provided by primary health care is family planning. Family planning refers to a conscious effort by a couple to limit or space births, or to avoid pregnancy when the desired family size has been achieved through the use of contraceptive methods. The Nigerian Primary Health Care sector, however, has faced a ‘brain drain’ of skilled health professionals, weak health facilities and systems, poor remuneration, frequent strike actions and corruption. These factors have combined to cripple Nigerian's health care sector,^5^ thus leaving a gap that continues to widen. Another factor is that states and Local Government Areas (LGAs) are not performing their respective responsibilities in managing and allocating resources to primary health care,^6^ or in terms of adequate health information on identified gaps, and updating documentation.

Where primary health care centres do exist, they lack the means to carry out home visits, especially in the rural, underserved and hard-to-reach communities. This is against the backdrop of the fact that 53% of Nigeria's total population resides in rural areas.^7^ As a consequence, information and knowledge on the need to access reproductive health services, often do not reach community members. This poor health-seeking behaviour has been associated with low literacy levels, myths, rumours, beliefs and misconceptions about contraceptives, a lack of access to contraceptives and a poor attitude of health care providers towards clients.

Programmes designed to reduce maternal mortality in Nigeria are designed as stand-alone, vertical programmes. Chilundo and Aanestad have stated that, often interventions designed as vertical programmes have been documented not to have yielded the much desired results.^8^ Even when programme designs are integrated, they do not carefully assess socio-cultural dynamics at the household level or develop interventions to build on the roles and strategies of other key household and community actors.^9^

Hence, it is imperative that community members are provided with correct and factual information and knowledge about contraceptives and its importance to maternal and child health. One key way of actualising this, is through the use of community health workers.

WHO (World Health Organisation) 1989,^10^ defines Community health workers as ‘members of the communities where they work, who should be selected by the communities, answerable to the communities for their activities, supported by the health system but not necessarily a part of its organisation, and have shorter training than professional workers’. In providing family planning services they often are referred to as Community Based Distribution Agents.

Community based distribution agents (CBDA) may operate out of health posts or depots, or may visit women in their homes. They may be volunteers, or may be paid a sales commission or a salary. Community Based Distribution (CBD) reduces the costs of contraception, thereby extending use amongst clients who seek contraceptives but will not use services that are confined to clinical settings.

The use of CBDA in Reproductive Health, especially in the reduction of maternal mortality and increasing access to contraceptives has been utilized in Uganda, India and Nigeria, amongst others. Such programmes, if thoughtfully designed and implemented effectively, provide a solid foundation of knowledge and skills on health, as well as increase access to health services in the communities. It is on this premise that this intervention was conceptualised.

We adopted the CBD approach to test the effectiveness of the agents in improving access to Sexual and Reproductive Health (SRH) information and services in 10 underserved communities in 5 Nigerian states. It is trusted that this intervention will contribute in part to the national effort to reduce the Maternal Mortality Ratio.

### Research significance

At present, 63% of women in developing countries use some method of family planning.^10^ Despite this increase, Nigeria still has an unmet need of 20% for family planning.^2^ Furthermore, a recent analysis shows that family planning is amongst a handful of feasible, cost-effective interventions that could impact immediately on maternal mortality in a lowresource setting.^11, 12^ The relationship between contraceptive use is directly proportional to maternal mortality reduction.^13^

Family planning offers a host of additional health, social and economic benefits:

- it can help to reduce infant mortality
- slow the spread of Human immunodeficiency virus (HIV) and AIDS
- promote gender equality
- reduce poverty
- accelerate socio-economic and healthy environment development.

It is, however, extremely challenging to make family planning information and services accessible to people living in rural, hard-to-reach areas.

Hence this study sought to use trained community members in providing family planning information and non-prescriptive family planning commodities, and make referrals to the closest primary health centre for prescriptive family planning commodities. This showcases the actualisation of one of the basic principle upon which the primary health care approach is hinged, which is community mobilisation, ownership and sustainability.

Also it demonstrated the contribution of CBDA to increasing community members’ access to family planning information and services.

## Ethical considerations

Ethics committee of the Association for Reproductive and Family Health reviewed and approved the proposal. The proposal was also submitted to the Project states Ministry of Health for review and acceptance. Participation was voluntary with confidentiality assured, and respondents received detailed information on the objective of the study. Verbal consent was obtained from participants before questionnaires were administered. Individual identifiers such as names and addresses were not included in the data collection instrument and thus collected data could not be linked to any of the respondents.

## Methods

### Setting

The Association for Reproductive and Family Health (ARFH) Ibadan, Nigeria is a non-governmental, non-profit organisation with a vision to improve the reproductive health status of men, women and young people in Nigeria and elsewhere in Africa. ARFH implemented a 3-year intervention (September 2005 to September 2008) with financial support from the United Nations Population Fund (UNFPA), in five Nigerian States namely, Bauchi and Gombe states (North-east), Plateau state (North-central), Edo state (South-south) and Ogun state (South-west). The geographic spread of the intervention is informed by a high maternal mortality rate and a low contraceptive prevalence in the selected states. In addition, each of the project states was supported by the United Nations Population Fund. In each of the selected states, the project was implemented in two LGAs as a pilot project that could be expanded or replicated in other LGAs in the states.

The intervention employed the Community Based Delivery (CBD) of non-prescriptive family planning services and the treatment of minor ailments in 10 project LGAs in the five states.

### The intervention

In the five project states, ARFH partnered with 10 indigenous non-governmental organization agents (NGOs) who had the mandate to coordinate CBD activities in each of the 10 project LGAs. The criteria for selection of two LGAs per state were rural location, underserved regions, low contraceptive prevalence rates and the willingness of the community members to participate in the project by recruiting or nominating volunteer community members as potential CBD agents.

Advocacy was a key strategy deployed on the project to facilitate an enabling environment for the project. A stakeholders’ workshop was also conducted in each state to sensitise and to enlist the support of the community members, as well as religious, community and opinion leaders, and policy makers.

Three training programmes were launched as a key component of the intervention:

- a 5-day Training of Trainers (TOT)
- a 5-day Management-skill and Supervisory-skill training for 50 participants from the selected partner NGOs and primary health care Service Providers
- a 16-day training of 250 volunteer community members as community based RH and FP service providers.

The CBD agents were trained to provide integrated services on the treatment of minor ailments and the provision of non-prescriptive family planning services, especially dual protection methods and pills, as well as counselling on HIV and AIDS. The CBD agents utilised the skills acquired during the capacity-building training to increase health literacy, to promote behaviour change and to generate demand for services in their communities. Refresher trainings were held to maintain the efficiency of the CBDA in providing quality RH and FP services. The CBDA were also linked to primary health care clinic staff that provided facilitated supervision and served as referral points for prescriptive family planning methods and treatment of other health conditions.

Non-prescriptive family planning commodities, anti-malarial drugs, multivitamins, analgesics and oral rehydration salts were provided to the trained agents for immediate commencement of client-friendly and quality services. These services were provided by the CBDA at a fixed rate in all the project states (Table 1), and the money generated enabled agents to obtain a resupply of commodities at the PHC clinic. The CBDA retained 25% of the fee, whilst the remaining 75% was remitted to the implementing NGOs for the resupply of drugs and commodities. The NGOs also provided a stipend to augment the 25% retained fee by the CBD to meet their transportation needs. Pictorial easy-to-use MIS forms were provided to the CBDA to document their activities.

**TABLE 1:** Fees charged by community based distribution agents for FP services and treatment of minor ailments.

Type of services	₦†	$†
Pack of Male Condom	15.00	0.10
Pack of female condom	20.00	0.13
Oral contraceptive pills	25.00	0.17
Malaria treatment for children	45.00	0.30
Malaria treatment for adults	65.00	0.43
Oral rehydration salts	20.00	0.13

*Source*: Authors’ original data

CBD, Community Based Distribution.

₦, Naira (monetary unit); $, US Dollar.

† Exchange rate of ₦150 to $1.

Male involvement was a crucial component of the intervention that was explored. The proficiency of some male CBDA was enhanced by using diverse Behavioural Change Communication (BCC) strategies, so that they could act as change agents amongst their male peers. Other BCC approaches adopted on the intervention to generate demand for services were the conduct of monthly outreach and edutainment activities by all the CBDA, the production and distribution of various IEC materials, sensitization seminars for journalists, and radio jingles which were aimed at increasing access to sexual reproductive health information and services through the mass media.

A laid-down performance monitoring and evaluation structure was established. The intervention was monitored monthly at four levels:

- The NGOs supervised the activities of the PHC clinic staff and CBDA at the first level.
- The PHC clinic personnel supervised and resupplied commodities to the CBDA at the second level.
- At the third level, ARFH personnel conducted monitoring and supervision quarterly.
- The UNFPA National Programme officer was involved in the final level of the monitoring to assess the progress of the project.

### Evaluation design

A Needs Assessment and Situation Analysis (NASA) survey was conducted amongst community members in the five project states in September 2005, as a means of documenting benchmark information for assessing the progress of the project, and to generate necessary information that will be useful in the planning and implementation of the CBD project.

Mid-way into the project implementation, it was deemed necessary to conduct a midterm evaluation to measure project progress and performance. The exercise conducted in December 2006 in the five project states, employed both quantitative (structured questionnaires) and qualitative research methods. Components of the surveys included community surveys and focus group discussions for community members. The questionnaire and focus-groupdiscussion modules for community members on the other hand, were designed to elicit information on their sociodemographic characteristics, perceived health problems, perceived causes of maternal death, utilisation of health facilities, utilisation of family planning services and their opinion about CBDA service delivery.

A final evaluation was also conducted in September 2008 to assess project outcomes in order to document the experiences of CBDA on the project. The questionnaire module for CBDA was designed to extract information from trained CBDA on issues such as training experience, service delivery, documentation and referral activities, as well as implementation challenges.

## Results

### Findings from the community based distribution agents

A total of 117 CBDA (53 male agents and 64 female agents) from the five intervention states were interviewed. Their activities were carried out in 10 different local government areas including Barkin Ladi, Bassa, Bogoro, Ganjuma, Yamaltu Deba, Funakaye, Ifo, Etsako Central and Uhunnwode. They covered 46 different wards and 69 communities. The distribution of the interviewed CBDA in the various geopolitical zones were documented (Table 2). Their occupation varied from farming (44%) to civil service (20.5%), housewives (14.5%), trading (11%), artisans (2.6%) and others (6.9%).

**TABLE 2:** Distribution of community based distribution agents In Project States.

Geopolitical Zones	State	Local government	%
North-Central	Plateau	Barkin Ladi Bassa	12.0 9.0
North- West	Bauchi	Bogoro	10.0
	Gombe	Ganjuma Yamaltu Deba Funakaye	9.0 10.0 10.0
South-West	Ogun	Ifo Ogun Waterside, Abigi	9.0 8.5
South-South	Edo	Etsako Central Uhunnwode	8.5 12.0

*Source*: Authors’ original data

### Training

An analysis of the administered questionnaires revealed that CBDA were trained to conduct out activities such as awareness creation on reproductive health and HIV and AIDS issues, referral of community members to hospitals or clinics, counselling, follow-up, immunisation, drug supply, home visits, awareness creation on environmental sanitation, as well as record keeping. All the trained CBDA reported that they were provided with kits containing drugs to treat minor ailments and to provide non-prescriptive family commodities commodities.

### Service provision

The CBDA provided services in different places in the communities, including the churches, mosques, palaces, schools, motor parks, primary health care centres, hotels and homes of community members. The fact that CBDA were able to increase access to services in diverse non-clinical outlets was expressed as follow by some of the CBDAinterviewed:

‘My highest service and sales as CBD agent comes from condom promotion and my clients are mostly my church members. Most of the condom sales were made after church programmes to the church members.’

‘I specialise in selling male condom(s) at drinking joints in the evenings. I have recorded a lot of sales through this strategy and my husband does not mind my involvement in such activities.’

‘I am a member of a Catholic Church in my community and I have presented health talk on family planning (including condoms and pills) to the whole church congregation on two occasions.’

At the final evaluation, findings revealed that the 250 trained CBDA had provided family planning services to 875 663 clients. A total of 36 145 clients had received oral pills, 834 257 had received male condoms, 85 had received female condoms and 5176 clients had been trained and adopted Natural Family Planning methods. In addition, 77 380 had received treatment for minor ailments, 150 285 were reached with RH information, 42 098 were referred for treatment and 67 362 male clients were reached by the male advocates with RH information. The Couple year of protection (CYP), which is the estimated protection provided by contraceptive methods during a 1-year period,^10^ was calculated and the total for all the family planning methods was 32 807.71 (Table 3).

**TABLE 3:** Number of family planning methods utilized by the clients and the Couple Year of Protection (CYP).

Family planning method	*n‡*	*n§*	CYP per unit	Total CYP
Oral contraceptive pills	36 145	36 145	15 cycles per CYP	2410
Male condoms†	834 257†	3 337 028†	120 units per CYP	27809
Female condoms	85	85	120 units per CYP	0.71
Natural Family Planning method	5176	5176	2 CYP per trained, confirmed adopter	2588

**Total**	**875 663**	-	**-**	**32 807.71**

*Source*: Authors’ original data

† For male condoms, number of users was multiplied by 4 because each client who received a strip of male condoms had 4 units each.

‡ *n*, number of Users.

§ *n*, number of Cycles/Unit.

### Family planning commodities and drug supply

The majority (96%) of the CBDA reported that a drugrevolving system was in place to ensure that drugs and commodities were always available for distribution. According to them, stock-out of commodities was avoided by ensuring that a revolving scheme was maintained (58%), making requisitions before drugs were completely out of stock (31%), ensuring that the drugs were properly stored (10%), and enlisting the supervisor's support (0.9%). Although the majority (99%) reported that the drug-revolving system was efficient, further probing revealed that approximately onethird (33%) only, of the CBDA, had ever experienced drugs and family planning commodities out of stock. Strategies employed to overcome drug stock-out included conducting referrals (55%), reporting to field supervisors (34%), and encouraging clients to wait for a resupply (8%), and collecting from fellow CBDA (5%).

### Referral activities

CBDA stated that they provided treatment for malaria and diarrhoea. Ailments (apart from malaria and diarrhoea) that could not be treated by them were referred to the Primary Health Care Centre. The majority (94%) of the CBDA reported that they follow-up clients referred by them to ensure that they receive treatment from the health centres.

### Documentation of activities

According to the interviewed CBDA, activities were recorded in their MIS forms. An on-the-spot check of the MIS forms at final evaluation showed that (86%) of them had their record sheets with them, and that their activities were recorded in their worksheets. A few (14%) of the CBDA stated that they left their record sheets at home.

### Challenges faced by community based distribution agents

The majority (85%) reported that they faced challenges during the process of service delivery. These challenges (Table 4) included discrimination and misconception of community members about family planning (38%), inadequate financial support (14%), transportation problems (8%); inadequate IEC materials (6%), poor supervision (3%); poor education (6%) inadequate time (2%) to deal with the demand for injectable contraceptives (2%), lack of identification cards (0.9%), poor spousal support amongst prospective family planning users (4%), complaints of side effects (bleeding) amongst community members (3%), and fear of side effects amongst community members (7%). A few (15%) CBDA, however, reported that they did not face any challenges.

**TABLE 4:** Challenges faced by community based distribution Agents (*N* = 99).

Challenges faced by CBD agents	%
Discrimination and misconception of community members	38
Inadequate financial support	14
Transportation problems	8
Inadequate IEC materials	6
Poor education	6
Poor spousal support	4
Complaints of side effects	3
Fear of side effects	7
Poor supervision	3

*Source*: Authors’ original data

*N*, total number of participants.

CBD, Community Based Distribution.

### Findings from Community Members

Community members were interviewed to assess their perception of common health problems in their community, their opinion about the CBDAs’ services and their utilization of family planning services. Findings at baseline were compared with findings observed after CBDA provided services to their respective communities.

### Socio-demographic characteristics

More than three-fifths of interviewed community members at both surveys were female; 68% at baseline and 70% at midterm evaluation. At both surveys, a greater proportion of interviewed members were aged 25–34 and the majority was married.

At least 20% of interviewed community members at both surveys were not educated, and for a significant proportion (66.5% at baseline and 45% at midterm), their income was less than ₦10, 000 per month. The socio-demographic characteristics of respondents at both baseline and final evaluation have been detailed (Table 5).

**TABLE 5:** Socio-demographic characteristics of community members (*N* = 579).

Socio-demographic Characteristics	Intervention (*N* = 579)	

	Before	After
**Sex**		
Male	32.0	30.0
Female	68.0	70.0
**Age**		
< 20	5.9	6.1
20–24	15.9	13.8
25–29	20.8	20.8
30–34	18.4	23.8
35–39	14.1	14.4
40–49	19.3	14.7
≥ 50	3.5	3.5
No response	2.1	2.8
**Marital status**	89.1	5.8
Married	7.6	90.2
Single	3.3	4.0
Other	3.3	4.0
**Type of Marriage**	*n* = 516	*n* = 514
Monogamy	65.0	72.0
Polygamy	35.0	28.0
**Religion**		
Christian	56.0	56.0
Islam	41.0	41.7
Traditional	3.0	2.3
**Income**		
≤ ₦10 000	66.5	45.2
₦10 000–₦19 999	21.2	16.0
≥ ₦20 000	3.6	7.0
Not disclosed	8.7 3	1.9
**Educational Qualification**		
No schooling	20.4	28.2
Adult Education	7.9	9.5
Primary	28.2	14.5
Secondary	32.5	16.1
Tertiary	11.1	30.6
Not disclosed	-	1.1

*Source*: Authors’ original data

₦, Naira (monetary unit); *N*, total number of participants.

### Perceived health problems

**FIGURE 1:**
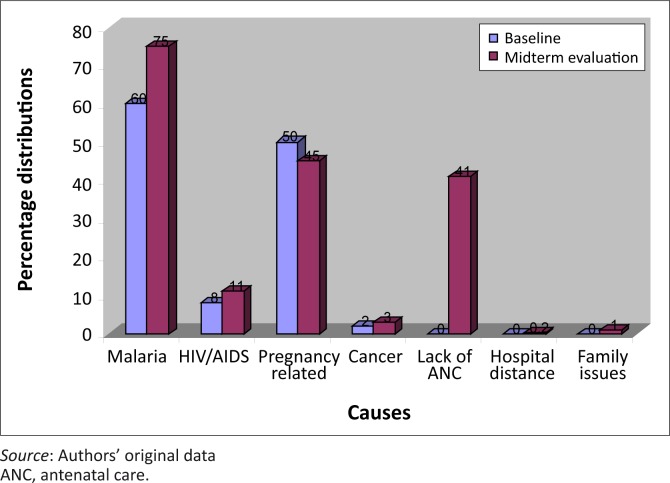
Common causes of maternal deaths as reported by community members.

The majority of the respondents reported malaria as the most common health problem in project communities at both baseline and midterm. Furthermore, when asked to identify the most common causes of maternal deaths, malaria was the most cited cause at both baseline and midterm evaluation. Other causes of maternal deaths include pregnancy-related causes such as haemorrhage, infection, unsafe abortions, hypertensive disorders, obstructed labour and anaemia, lack of ante-natal care, and HIV and AIDS, amongst others (Figure 1).

#### Utilization of health facilities

**FIGURE 2:**
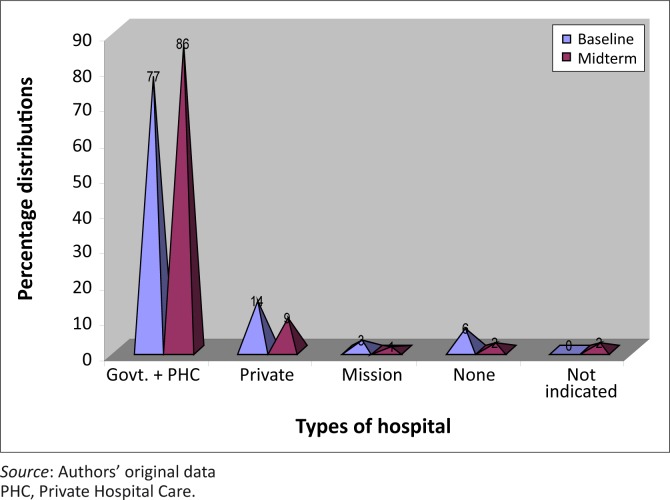
Type of health facilities utilized by community members.

When probed about the available health facilities in project communities, government hospitals (including PHC clinics) were the most frequently mentioned available health facility in the project communities. They were also the most frequently utilised health facilities before and after intervention. There was about 50% increase in the utilisation of government facilities after the intervention. Other health facilities utilised by community members include private clinics and mission hospitals. The chart below (Figure 2) gives a description of the types of facilities utilised by community members.

#### Opinion about community based distribution agents’ service delivery

A large proportion of respondents (83%) reported that they knew the CBDA and were aware of their activities in their communities. About two-thirds reported that they had been counselled by CBDA, more than half stated that they were given IEC materials, 46% said they had received treatments for minor ailments, whilst 28% reported that they had been referred by CBDA. The overall assessment of 80% of the interviewed community members was that community members benefited by virtue of CBDA, through their provision of information and services (about 50%), condom distribution (32%) and pill distribution (29%), as well as outreach activities (about 50%).

#### Community members’ utilisation of family planning services

**FIGURE 3:**
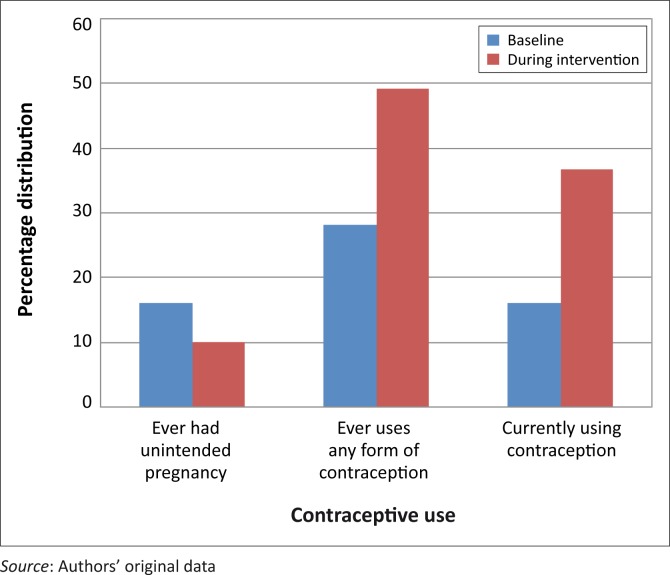
Community members’ use of contraception before and during Intervention.

A comparison of community members’ utilisation of family planning commodities at baseline and midterm evaluation showed an increase in the utilisation of family planning services. The proportion of community members who *had* utilised family planning commodities increased from 28% at baseline to 49% at midterm evaluation, whilst the proportion of those who were current users of family planning commodities increased from 16% at baseline to 37% at midterm evaluation. Furthermore, the proportion of those who had ever had unintended pregnancies, dropped from 16% to 10% (Figure 3).

At both surveys, community members were questioned to identify family planning methods known and utilised (Table 6). A comparison of knowledge before, and during intervention, demonstrated an increase in knowledge of common family planning methods, including male and female condoms, injectables and pills. It was also observed in a number of clients that had ever used condoms and pills, although the current use in all cases was observed to be lower than ‘ever use’.

**TABLE 6:** Contraceptive use amongst Community Members before and after Intervention.

Contraceptive methods	Intervention					

	Before			During		
	
	Knowledge	Ever Use	Current Use	Knowledge	Ever Use	Current Use
Male Condom	71.0	6.0	2.8	84	30.0	12.0
Female Condom	33.5	0.8	0.8	47	8.0	0.7
Injectables	70.0	11.0	3.0	80	10.5	6.0
Pills	71.0	12.0	4.0	88	29.0	14.0
IUCD	41.0	2.0	0.6	36	1.7	3.0
Implants	20.0	0.8	0.6	17	0.5	0.1
Female sterilization	29.0	0.8	0.8	19	-	0.1
Male sterilization	18.0	0.1	-	18	-	-
Emergency contraceptives	19.5	1.2	0.5	13	2.2	1.0
Natural methods	39.0	7.0	3.6	40	7.0	3.0
Traditional methods	39.0	4.0	0.5	31	4.0	2.0

*Source*: Author's original data

IUCD, intrauterine contraceptive device.

## Discussion

The Reproductive Health status in Nigeria is sub-standard as reflected in the high maternal morbidity and mortality rate, the high infant mortality rate, the low contraceptive prevalence rate, the poor status of adolescent reproductive health, as well as the prevalence of STIs and HIV and AIDS, especially amongst young people.^11^

The factors underlying the poor reproductive health situation in Nigeria manifest on several levels from individual to community and health facility levels. Some women are denied access to care because of lack of information regarding the right decisions and harmful traditional practices. Other contributory factors are largely attributable to the failure of the health systems. In Nigeria, women, especially those in the rural areas, lack access to essential reproductive health services because of factors such as unavailability of health care facilities and trained providers, poor road networks and transportation. Where services *are* available, utilisation is low. The utilisation of modern contraceptives, for example, is generally low in Nigeria. The CPR of 15% is amongst the lowest in sub-Saharan Africa. Rates vary widely, from 4% in the North-east to 13% in the North-central region and 32% in the South.^2^ Low CPR is reflected in the persistently high TFR and the unmet need for FP (20%) is still high. Factors associated with low contraceptive use include low levels of awareness, an irregular supply of contraceptives, poor method mix, low availability and accessibility to quality FP services. These are accentuated by a negative traditional perspective, and religious as well as socio-cultural beliefs and myths about contraceptive use.

Improvement of reproductive health is central to achieving the Millennium Development Goals on maternal health and child mortality, and to eradicate extreme poverty.^14, 15^ These goals require women to have access to safe and effective methods of fertility control. The promotion of family planning to avoid unintended pregnancies is central to the World Health Organization's (WHO's) efforts to improve maternal health, and is paramount to achieving the Millennium Development Goals.^16^ Increased access to, and voluntary use of contraceptives through community based initiatives, is therefore a key strategy in addressing this situation, especially in view of the health system failure.

Similar to results documented in this intervention, a study conducted by the Population Council in Mali demonstrated that community based distribution of reproductive health services can contribute to an increase in contraceptive usage.^17^. An analysis of this intervention in Mali revealed that there was an increase in contraceptive usage from 1% to 31% over a 2-year intervention period. This study also revealed a significant increase in knowledge and contraceptive usage after the introduction of the CBD intervention and this demonstrates a correspondence with several studies.^17–19^ Improvements in knowledge and contraceptive usage can be attributed to the enhanced effectiveness of the CBDAand the access of vulnerable hard-to-reach populations to services through diverse non-clinical outlets. This evidence suggests that further improvements in access and quality of services can increase the use of contraceptives even more.

Stimulating change in a community requires considerable sensitivity and patience, and must be supported by the community's leadership. Furthermore, significant RH and FP improvements in many communities cannot be realised without substantial changes in cultural and social norms, including the status of girls and women. Even in the most open-minded societies this type of change cannot be imposed from the outside, but must grow from within. According to Burket,^20^ the challenge is greater in traditional, conservative societies. The community leadership was involved in the implementation of the CBD intervention in the project communities. They had the mandate to select the CBDAand were also represented in the Project Advisory committee. This may be responsible for the acceptance of the CBDA by the community members.

The selection of CBDA by the communities and their social acceptability is crucial for the success of CBD programmes.^21^ It is particularly important considering the fact that community based programmes attempt to capitalise on social networks.^22^ This underscores the importance of ensuring that the process is community-driven, especially the selection of the CBDA and design of operations.^21^

Although the majority of CBDA reported that the drugrevolving system was functional, about a third had experienced a stock-out of the drugs and family planning commodities. This is similar to the findings of a study conducted by the Population Council and Planned Parenthood Association of Ghana which assesses CBD programmes in Ghana. A key strategy for reducing stock-out is to increase the frequency of supervision of the CBDAas well as improving the commodity logistics management system to ensure that the minimum stock of commodities are available to all agents at all times.^23^

The majority of CBDA reported that they encountered operational challenges during the process of service delivery. These challenges included inadequate financial support and transportation problems, poor spousal support amongst prospective family planning users, complaints of side effects by users acceptor and so forth. These views were also expressed by CBDA in the German Agency for Technical Co-operation (GTZ) assisted project sites in Ethiopia.^24^ These findings are very important for the design and implementation of a successful CBD programme and require the development and implementation of an effective model for mitigating these challenges. Although fundinglimitations and concerns about sustainability is an obstacle in many programmes, because salaries or monetary rewards for CBDA cannot be provided, they should be compensated at the least for time spent away from their families, fields, and other work.^20^ Based on ARFH's experience in implementing the CBD project, some of the essential features which can be incorporated in the design of any CBD intervention to address operational challenges and to enhance sustainability, include:

- performance based compensations which may be financial or otherwise
- recognition and certification of the CBDA
- an innovative transportation system (provision of motorcycles or tricycles which can be used in service delivery as well as a source of generating income)
- income-generating activities.

A key set-back in interventions designed by using the CDB approach, has been the inability to ensure sustainability. With this intervention, we involved the health authorities at both the State and LGAs of the five states in the design, implementation and evaluation of the intervention. This created a sense of belonging and ownership of the initiative by the Ministry of Health. Hence, following the end of funding for the intervention by UNFPA, the local government gladly accepted the CBDA as part of its health force and this was exemplified by the use of the CBDA for community health sensitisation during National Immunization Days and during outbreaks of epidemics in their communities.

A review of the activities of the CBDA in the sphere of providing non-prescriptive family planning commodities was carried out 2 years later. This review showed that of the 250 CBDA trained, 175 were still involved in providing information and service in the project communities.

## Conclusion

This study has demonstrated that community based distribution can play a critical role in enhancing access to reproductive health and family planning information and services. Strategies to enhance the effectiveness of CBD programmes will include community involvement in the selection of CBDA, a drug-revolving scheme, and regular update sessions for CBDA to improve their technical capacity, agent motivation, service integration, improved supervision and management information systems. Other important factors which can contribute to the success of CBD programmes include enhanced CBD clinic referral and improved commodity logistics management systems.

### List of Abbreviations

**Table:** 

**ARFH**	Association for Reproductive and Family Health
**CBD**	Community Based Distribution
**CBDA**	Community Based Distribution (Agent)
**FP**	Family Planning
**IEC**	Information, Education and Communication
**LGA**	Local Government Area
**MIS**	Management Information System
**NGOs**	Non-Governmental Organisations
**RH**	Reproductive Health
**STIs**	Sexually Transmitted Infections
**UNFPA**	United Nations Population Fund
